# Interferon-Stimulated Gene (ISG)-Expression Screening Reveals the Specific Antibunyaviral Activity of ISG20

**DOI:** 10.1128/JVI.02140-17

**Published:** 2018-06-13

**Authors:** Junjie Feng, Arthur Wickenhagen, Matthew L. Turnbull, Veronica V. Rezelj, Felix Kreher, Natasha L. Tilston-Lunel, Gillian S. Slack, Benjamin Brennan, Elina Koudriakova, Andrew E. Shaw, Suzannah J. Rihn, Charles M. Rice, Paul D. Bieniasz, Richard M. Elliott, Xiaohong Shi, Sam J. Wilson

**Affiliations:** a MRC-University of Glasgow Centre for Virus Research, Glasgow, United Kingdom; b Laboratory of Virology and Infectious Disease, Center for the Study of Hepatitis C, The Rockefeller University, New York, New York, USA; c Laboratory of Retrovirology, The Rockefeller University, New York, New York, USA; d Howard Hughes Medical Institute, The Rockefeller University, New York, New York, USA; University of Southern California

**Keywords:** ISG20, ISGs, bunyavirus, innate immunity, interferons, restriction factor

## Abstract

Bunyaviruses pose a significant threat to human health, prosperity, and food security. In response to viral infections, interferons (IFNs) upregulate the expression of hundreds of interferon-stimulated genes (ISGs), whose cumulative action can potently inhibit the replication of bunyaviruses. We used a flow cytometry-based method to screen the ability of ∼500 unique ISGs from humans and rhesus macaques to inhibit the replication of Bunyamwera orthobunyavirus (BUNV), the prototype of both the Peribunyaviridae family and the Bunyavirales order. Candidates possessing antibunyaviral activity were further examined using a panel of divergent bunyaviruses. Interestingly, one candidate, ISG20, exhibited potent antibunyaviral activity against most viruses examined from the Peribunyaviridae, Hantaviridae, and Nairoviridae families, whereas phleboviruses (Phenuiviridae) largely escaped inhibition. Similar to the case against other viruses known to be targeted by ISG20, the antibunyaviral activity of ISG20 is dependent upon its functional RNase activity. Through use of an infectious virus-like particle (VLP) assay (based on the BUNV minigenome system), we confirmed that gene expression from all 3 viral segments is strongly inhibited by ISG20. Using *in vitro* evolution, we generated a substantially ISG20-resistant BUNV and mapped the determinants of ISG20 sensitivity/resistance. Taking all the data together, we report that ISG20 is a broad and potent antibunyaviral factor but that some bunyaviruses are remarkably ISG20 resistant. Thus, ISG20 sensitivity/resistance may influence the pathogenesis of bunyaviruses, many of which are emerging viruses of clinical or veterinary significance.

**IMPORTANCE** There are hundreds of bunyaviruses, many of which cause life-threatening acute diseases in humans and livestock. The interferon (IFN) system is a key component of innate immunity, and type I IFNs limit bunyaviral propagation both *in vitro* and *in vivo*. Type I IFN signaling results in the upregulation of hundreds of IFN-stimulated genes (ISGs), whose concerted action generates an “antiviral state.” Although IFNs are critical in limiting bunyaviral replication and pathogenesis, much is still unknown about which ISGs inhibit bunyaviruses. Using ISG-expression screening, we examined the ability of ∼500 unique ISGs to inhibit Bunyamwera orthobunyavirus (BUNV), the prototypical bunyavirus. Using this approach, we identified ISG20, an interferon-stimulated exonuclease, as a potent inhibitor of BUNV. Interestingly, ISG20 possesses highly selective antibunyaviral activity, with multiple bunyaviruses being potently inhibited while some largely escape inhibition. We speculate that the ability of some bunyaviruses to escape ISG20 may influence their pathogenesis.

## INTRODUCTION

There are over 350 known viruses in the Bunyavirales order (collectively known as bunyaviruses) that were recently reclassified into nine families: Feraviridae, Fimoviridae, Hantaviridae, Jonviridae, Nairoviridae, Peribunyaviridae, Phasmaviridae, Phenuiviridae, and Tospoviridae (1). Most bunyaviruses are carried and transmitted by arthropods, such as mosquitoes, ticks, sand flies, or thrips, except the hantaviruses (Hantaviridae), which are typically transmitted via infectious aerosols usually originating from rodent excreta (2). Several bunyaviruses can cause severe human diseases, such as La Crosse (LACV) and Oropouche (OROV) orthobunyaviruses (Peribunyaviridae family), Hantaan (HTNV) and Sin Nombre orthohantaviruses (Hantaviridae family), severe fever with thrombocytopenia syndrome (SFTSV) and Rift Valley fever (RVFV) phleboviruses (Phenuiviridae family), and Crimean-Congo hemorrhagic fever orthonairovirus (CCHFV) (Nairoviridae family) (2). Importantly, bunyaviral emergence and reemergence represent continuous threats to global health and prosperity, and bunyaviruses might have higher zoonotic potential than that of many other viruses (3). Bunyamwera orthobunyavirus (BUNV), the prototype virus of the Peribunyaviridae family and the Bunyavirales order, remains an important research model for many significant bunyaviral pathogens.

Like most viruses in the Peribunyaviridae family, BUNV possesses a tripartite negative-sense RNA genome comprised of large (L), medium (M), and small (S) genome segments. The S segment encodes the nucleocapsid (N) protein and the nonstructural protein NSs in overlapping reading frames. The M segment encodes a viral glycoprotein precursor (in the order Gn-NSm-Gc), and the L segment encodes the RNA-dependent RNA polymerase (2). The glycoprotein precursor is proteolytically cleaved into two mature viral membrane glycoproteins (Gn and Gc) and a nonstructural protein (NSm) by the host signal peptidase and signal peptide peptidase (4). Bunyaviruses replicate in the cytosol and assemble and bud at membranes of the Golgi complex (2). During genome replication, each genome segment serves as an RNA-dependent RNA polymerase template for the generation of positive-sense mRNA and antigenomic RNA (cRNA). The cRNA subsequently acts as a template for the generation of nascent genomic RNA (gRNA). Thus, bunyavirus replication involves at least nine distinct RNA species (2).

Following infection, bunyaviruses are sensed by the host. For example, the RNA genomes of orthobunyaviruses and phleboviruses carry uncapped 5′ triphosphate (5′-pppRNA) ends and short double-stranded RNA (dsRNA) structures, which can be sensed by the cytoplasmic RNA helicase, RIG-I (retinoic acid-inducible gene I) (5–7). Pattern recognition frequently results in the secretion of type I interferons (IFNs), which modulate multiple immune processes and place cells in an “antiviral state,” impeding the infection and replication of viruses (8). IFNs are known to inhibit bunyaviruses both *in vitro* and *in vivo* (9–16). Indeed, IFNs likely play a key role in constraining bunyavirus replication and pathogenesis, as many short-lived asymptomatic infections can become severely pathogenic when the IFN defenses of the host are compromised (17–22). Moreover, the importance of host IFN responses in combatting bunyaviral infection is underscored by the multitude of strategies that bunyaviruses employ to counteract host IFN responses. The NSs proteins of many bunyaviruses can be major virulence factors and act as potent IFN antagonists (23–25). These divergent NSs proteins utilize multiple strategies to inhibit host IFN responses (25, 26), including suppressing host mRNA transcription (25, 27, 28), blocking pattern recognition (11, 29, 30), or even disrupting type I IFN signaling by sequestering STAT1 and STAT2 into inclusion bodies (31).

Although bunyaviruses are potently inhibited by IFNs *in vitro* and this inhibition likely helps to define bunyaviral pathogenesis, only a few IFN-stimulated genes (ISGs) have been ascribed antibunyaviral activity so far (32–36). In this study, using a flow cytometry-based gain-of-function screening assay, we considered the ability of 488 unique human and macaque ISGs to inhibit the prototypical bunyavirus (BUNV). Here we show that the antiviral exonuclease ISG20 (37) has broad-spectrum antiviral activity against multiple bunyaviruses. Similar to that against known targets of ISG20, the antiviral effect is dependent upon functional exonuclease activity (37–41). Using *in vitro* evolution, we selected an ISG20-resistant BUNV and showed that viral resistance maps to multiple genome segments. Importantly, even low levels of endogenous ISG20 expression potently inhibited BUNV, whereas some bunyaviruses effectively escaped inhibition by ISG20. These data suggest that ISG20 may play a key role in the host response to bunyaviral infection and that sensitivity/resistance to this factor might therefore influence bunyaviral pathogenesis.

## RESULTS

### BUNV-EGFP completes a single round of infection in MT4 cells.

**FIG 1:**
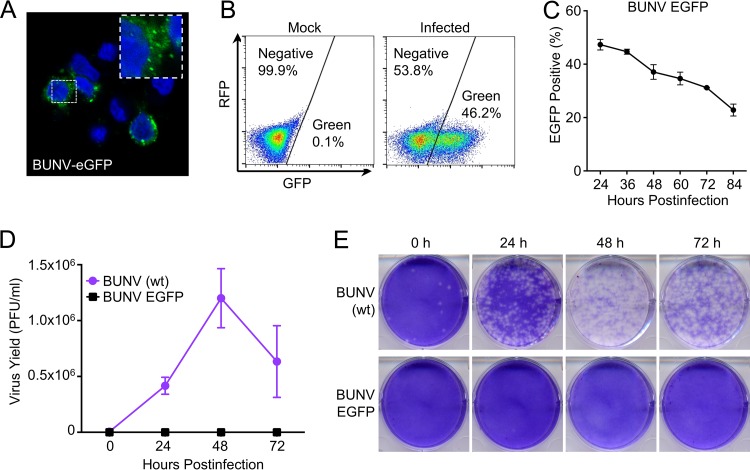
BUNV-EGFP completes a single round of infection in MT4 cells. (A) A typical confocal microscopy field of MT4 cells infected with BUNV-EGFP and stained with DAPI (4′,6-diamidino-2-phenylindole). (B) Pseudocolored flow cytometry dot plots of MT4 cells infected or not infected with BUNV-EGFP. (C) Growth curve for BUNV-EGFP on MT4 cells. MT4 cells were infected with BUNV-GFP at an MOI of 5 (determined using Vero cells). (D) Growth kinetics of wt BUNV and BUNV-EGFP in MT4 cells. MT4 cells were infected with wt BUNV or BUNV-EGFP, using the same MOI. At different time points, the supernatant was harvested and the infectious titer determined using Vero cells. (E) Plaques of wt BUNV and BUNV-EGFP (on Vero cells) produced by MT4 cells.

We previously reported a “multicycle” screen searching for host factors capable of attenuating BUNV replication. Under these conditions, IFITM3 and IRF1 were identified as modest inhibitors of BUNV replication (42). Because BUNV is highly sensitive to inhibition by type I IFN *in vitro*, we hypothesized that additional ISGs with anti-BUNV activity likely exist. We recently expanded our ISG library to include ∼350 additional ISGs from rhesus macaques (43), increasing our total library size to 488 unique ISGs (∼25% more genes). This encouraged us to search again for interferon-induced effectors that inhibit BUNV. In order to use arrayed ISG-expression screening (42–45) to identify ISGs with anti-BUNV activity, we needed to select a cell line that could easily be transduced with ISG-encoding lentiviral vectors and be susceptible to subsequent infection with BUNV. We previously used human MT4 T cells for this screening approach (43, 44), so we examined whether these cells were susceptible to BUNV infection. We utilized recombinant BUNV-EGFP, in which enhanced green fluorescent protein (EGFP) is fused with the N terminus of a truncated Gc glycoprotein (46), as this reporter virus can easily be used for flow cytometry-based screening assays. Interestingly, although MT4 cells could readily be infected with BUNV-EGFP (Fig. 1A and B), this virus appeared to be unable to spread throughout the culture, and the percentage of infected cells decreased over time (Fig. 1C). The lack of propagation in MT4 cells appeared to be specific to the recombinant GFP-tagged virus, as wild-type (wt) BUNV replicated efficiently (reaching titers of over 10^6^ PFU/ml at 48 h postinfection [hpi]), whereas no PFU were produced with BUNV-EGFP at an equivalent multiplicity of infection (MOI) (Fig. 1D and E). The exact mechanism that prevented BUNV-EGFP propagation in MT4 cells is unknown, but the formation of abundant virus-like GFP puncta (Fig. 1A) suggested that BUNV-EGFP may efficiently express multiple viral genes and complete much of the viral life cycle in these cells. Construction of BUNV-EGFP required deleting 350 residues from the N terminus of Gc to accommodate the GFP moiety. It is therefore possible that the N terminus of Gc is essential for the efficient generation of infectious BUNV progeny in MT4 cells but is dispensable in other contexts (46, 47).

### ISG-expression screening reveals candidate genes with anti-BUNV activity.

**FIG 2:**
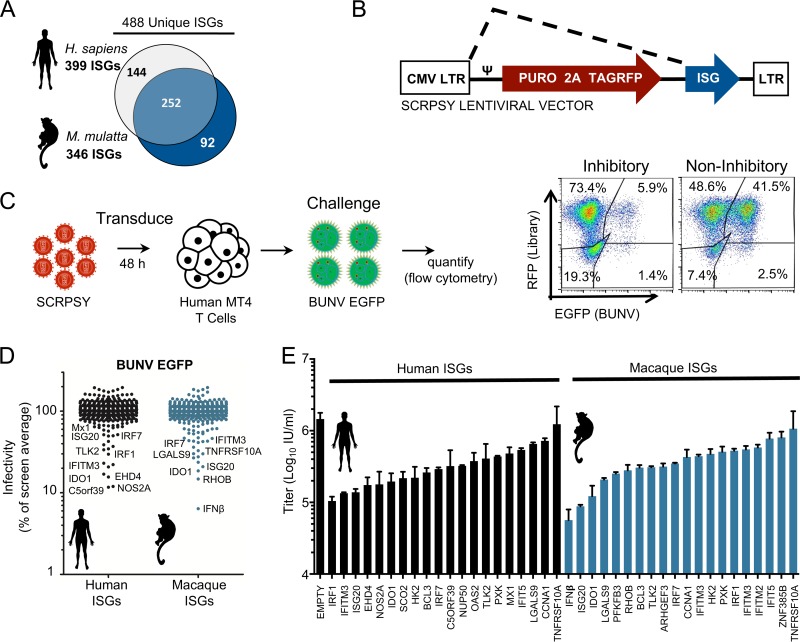
ISG-expression screening reveals novel candidate genes with anti-BUNV activity. (A) Schematic representation of H. sapiens and M. mulatta ISG libraries. The libraries are in a 96-well plate format with one ISG per well. (B) Simplified schematic of the pSCRPSY-DEST lentiviral vector (the dotted line indicates mRNA splicing). (C) Schematic representation of the ISG screen. MT4 cells were transduced with the ISG libraries. After 48 h, cells were challenged with BUNV-EGFP, and the infectivity of BUNV-EGFP in ISG-expressing MT4 cells was quantified by flow cytometry at 48 hpi. (D) Results of the screen. Each dot represents one ISG. Infectivity measured for each ISG-expressing well was normalized to the average for the screen, which is indicated as 100%. (E) Confirmatory assays for the selected top 20 H. sapiens and M. mulatta candidate inhibitory ISGs. New lentiviral stocks independent of the primary screen were made and used to transduce MT4 cells. BUNV-EGFP infection was performed as in the primary screen. Titers are presented as numbers of GFP infectious units (IU) per milliliter and were calculated for triplicate wells.

The unanticipated single round of infection of BUNV-EGFP in MT4 cells enabled us to conduct a screen searching for ISGs that inhibit the early stages of the BUNV life cycle (after viral gene expression but before the genesis of infectious progeny). Arrayed ISG-expression screening involves exogenously expressing ISGs within target cells that are susceptible to infection by the virus of interest (43–45). Under screening conditions, not all the cells in a well will express the ISG. Viruses can therefore replicate efficiently in the ISG-negative cells, and abundant progeny virus can overwhelm a protective ISG in adjacent cells. Thus, “multicycle” ISG-expression screens can potentially underestimate the protective effect of some ISGs, and limiting the virus to a single round of infection may improve the identification of candidate antiviral factors. Thus, we took advantage of the late defect in BUNV-EGFP replication in MT4 cells to examine the ability of 488 different ISGs to inhibit a single round of BUNV infection (Fig. 2A to D). We considered ISGs from Homo sapiens and Macaca mulatta (43), as both primate species likely support BUNV infection (48, 49). The protective effect of each ISG was quantified using two-color flow cytometry (Fig. 2C), and the level of infection in the presence of each ISG was plotted as a percentage of the screen average (Fig. 2D).

Multiple ISGs conferred considerable protection from BUNV infection in the initial screen (Fig. 2D). Many of the candidate anti-BUNV ISGs were known restriction factors (such as ISG20, IFITM3, and Mx1). In addition, multiple ISGs (such as IRF1 and IRF7) likely conferred protection by engendering a polygenic antiviral state by stimulating IFN expression or interferon-stimulated response element (ISRE)-driven transcription (43).

To validate the screening results, independent lentiviral vector preparations were used to consider the ability of the 20 most protective human and macaque candidate antiviral ISGs to protect MT4 cells from titrated BUNV-EGFP challenge (Fig. 2E). This confirmed the ability of the exogenously expressed ISGs to confer protection from BUNV infection. Nine human ISGs and 5 macaque ISGs which conferred ≥5-fold protection were selected for further investigation (Fig. 2E; Tables 1 and 2).

**TABLE 1:** Results of confirmatory assays for H. sapiens ISGs

Rank	Gene symbol	Product name	Fold inhibition (ISGs/empty SCRPSY)
1	IRF1	Interferon regulatory factor 1	13.94188652
2	IFITM3	Interferon-induced transmembrane protein 3	10.81898208
3	ISG20	Interferon-stimulated exonuclease gene, 20 kDa	10.51226219
4	EHD4	EH domain-containing protein 4	8.337769295
5	NOS2A	Nitric oxide synthase 2a	8.142114105
6	INDO/IDO	Indoleamine-pyrrole 2,3-dioxygenase	7.431693179
7	SCO2	SCO cytochrome oxidase-deficient homolog 2	6.682186081
8	HK2	Hexokinase 2	6.600853655
9	BCL3	B-cell CLL/lymphoma 3	5.560601225
10	IRF7	Interferon regulatory factor 7	4.996490044
11	C5ORF39	Annexin A2 receptor	4.532151199
12	NUP50	Nucleoporin, 50 kDa	4.502720506
13	OAS2	2′,5′-Oligoadenylate synthetase 2	3.857183543
14	TLK2	Tousled-like kinase 2	3.560273013
15	PXK	PX domain-containing serine/threonine kinase-like protein	3.333094361
16	MX1	Myxovirus (influenza virus) resistance 1	3.019964468
17	IFIT5	Interferon-induced protein with tetratricopeptide repeats 5	2.697749553
18	LGALS9	Galectin 9, beta-galactoside-binding protein	2.222739332
19	CCNA1	Cyclin A1	2.017553608
20	TNFRSF10A	Tumor necrosis factor receptor superfamily, member 10a	1.181220011

**TABLE 2:** Results of confirmatory assays for M. mulatta ISGs

Rank	Gene symbol	Product name	Fold inhibition (ISGs/empty SCRPSY)
1	IFNβ1	Beta interferon 1	25.68764253
2	ISG20	Interferon-stimulated exonuclease gene, 20 kDa	16.51376275
3	INDO/IDO	Indoleamine-pyrrole 2,3-dioxygenase	11.89015349
4	LGALS9	Galectin 9, beta-galactoside-binding protein	6.99430233
5	PFKFB3	6-Phosphofructo-2-kinase/fructose-2,6-biphosphatase 3	5.758834317
6	RHOB	Ras homolog family member B	5.195047578
7	BCL3	B-cell CLL/lymphoma 3	4.754072894
8	TLK2	Tousled-like kinase 2	4.709891813
9	ARHGEF3	Rho guanine nucleotide exchange factor (GEF) 3, isotype	4.611043388
10	IRF7	Interferon regulatory factor 7	4.174521863
11	CCNA1	Cyclin A1	3.389544757
12	IFITM3	Interferon-induced transmembrane protein 3	3.301078884
13	HK2	Hexokinase 2	3.060345699
14	PXK	PX domain-containing serine/threonine kinase-like protein	2.855499432
15	IRF1iso3	Interferon regulatory factor 1, isotype	2.770775682
16	IFITM3	Interferon-induced transmembrane protein 3	2.64956607
17	IFITM2	Interferon-induced transmembrane protein 2	2.495300874
18	IFIT5	Interferon-induced protein with tetratricopeptide repeats 5	1.868064022
19	ZNF385B	Zinc finger protein 385B	1.802174105
20	TNFRSF10A	Tumor necrosis factor receptor superfamily, member 10a	1.363085906

### ISG20 has specific antibunyaviral activity.

**FIG 3:**
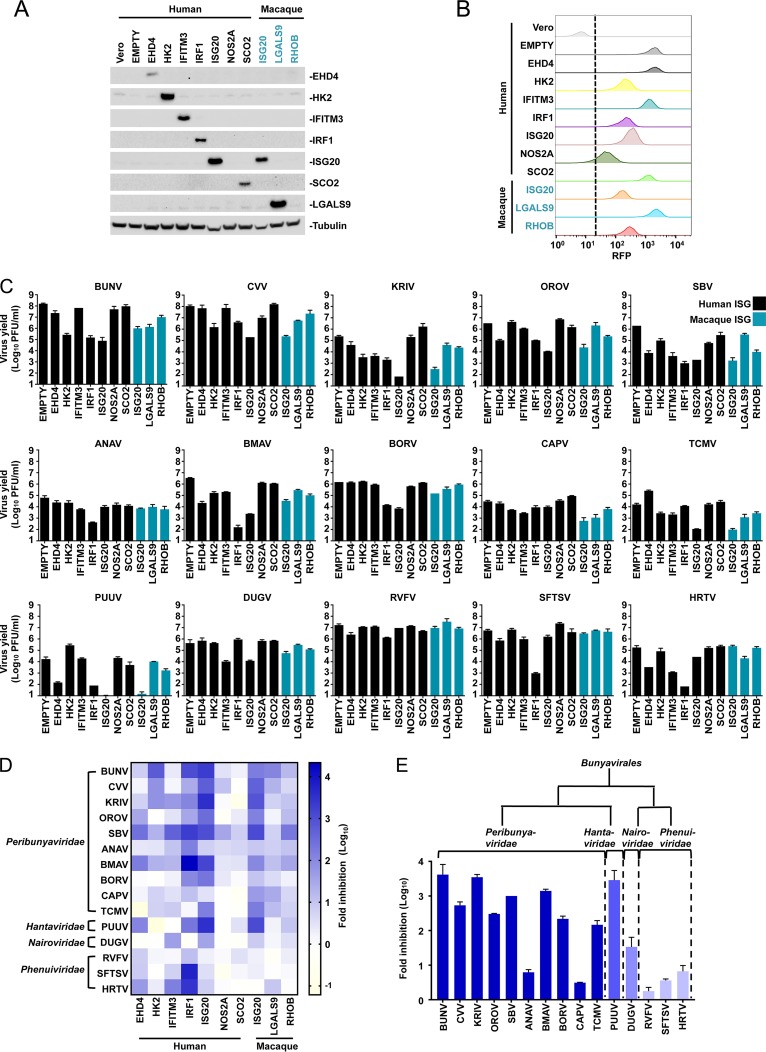
Antibunyaviral activities of candidate ISGs against a panel of divergent bunyaviruses. (A) ISG expression in 10 Vero-ISG-expressing cell lines in addition to “empty vector” and naive Vero cell controls was assessed using Western blotting (assays were not established for NOS2A and RHOB). (B) For the ISGs in panel A, lentiviral vector-encoded TagRFP expression was monitored by flow cytometry. (C) Infectious virus yields from 10 Vero-ISG-expressing cell lines. Cells were infected with 15 wild-type bunyaviruses representing four families. Supernatants were collected, and virus yields were determined on Vero or BHK-21 cells. The MOIs and time points of supernatant harvest were as follows: for BUNV, CVV, KRIV, SBV, DUGV, RVFV, and SFTSV, MOI of 0.001 and 72 hpi; for OROV, MOI of 0.001 and 48 hpi; for ANAV, BMAV, BORV, CAPV, and TCMV, MOI of 0.01 and 48 hpi; for PUUV, MOI of 0.1 and 48 hpi; and for HRTV, MOI of 0.01 and 72 hpi. EMPTY, Vero cells transduced with empty SCRPSY lentiviral stocks. (D) Heat map analysis (generated by GraphPad Prism 7) of the effects of 10 ISGs on the yields of 15 bunyaviruses. (E) Summary of human ISG20's antiviral effects on 15 bunyaviruses.

Many divergent viruses are classed as bunyaviruses (1, 2). To further investigate the antiviral spectra of the selected ISGs against bunyaviruses, we screened the ability of each ISG which conferred ≥5-fold protection from BUNV to inhibit a panel of 15 divergent bunyaviruses (including members of four distinct families). Our panel included viruses that can cause serious clinical infections, such as heartland phlebovirus (HRTV) and SFTSV, as well as agricultural pathogens, such as Schmallenberg orthobunyavirus (SBV). To compare these divergent viruses, we used Vero cells, which support the efficient propagation of many divergent viruses due in part to their defective IFNB1 gene (50). To ensure stable phenotypes, we established Vero cell clones that were transduced with vectors encoding human EHD4, HK2, IFITM3, IRF1, ISG20, NOS2A, or SCO2 in addition to clones modified to express macaque ISG20, LGALS9, or RHOB. Single-cell clones were selected that had BUNV-inhibitory profiles similar to those of the parental “bulk” population from which they were derived. We were unable to establish stable cell lines expressing IDO1, BCL3, or PFKFB3. The broad-spectrum antiviral activity of IDO1, through tryptophan depletion, is well documented (43), and persistent tryptophan depletion likely inhibited the generation of single-cell clones. We reasoned that the ability of BCL3 and PFKFB3 to perturb normal Vero cell physiology (preventing the generation of stable cell lines) made it less likely that these ISGs possessed direct anti-BUNV activity and more likely that the global cellular effect of overexpressing these genes reduced BUNV infectivity. Thus, we did not consider IDO1, BCL3, or PFKFB3 further. We confirmed the expression of eight ISGs by Western blotting (Fig. 3A). However, we were unable to establish Western blotting conditions that detected NOS2A or RHOB. We also assessed the transduction and clonality of each cell line by monitoring red fluorescent protein (RFP) expression from the lentiviral vector (Fig. 3B). We then conducted a miniscreen of the 10 remaining ISGs (7 human and 3 macaque ISGs) and assessed the virus yields for our panel of bunyaviruses in the presence of each ISG (Fig. 3C). Because one clone of each ISG was investigated, we used this miniscreen approach to select candidates for subsequent examination, and the miniscreen does not constitute a precise survey of ISGs that inhibit representative bunyaviruses. Moreover, NOS2A caused little inhibition of any of our panel of viruses. Without further investigation, it is impossible to discern whether NOS2A was poorly expressed or has limited antibunyaviral activity. Nonetheless, some patterns emerged, and the inhibitory effects of each ISG on our panel of bunyaviruses are summarized in the heat map in Fig. 3D.

As observed in previous studies, exogenous IRF1 expression initiated a gene expression program (43) that led to the inhibition of most bunyaviruses. IFITM3, a well-characterized restriction factor that inhibits the fusion of cellular and viral membranes (51), appeared to have broad-spectrum antibunyaviral activity. IFITM3 reduced the infectious yield and/or plaque area for most of the members of our panel of bunyaviruses. The magnitude of protection was variable, as IFITM3 potently inhibited several viruses (such as Kairi orthobunyavirus [KRIV], SBV, Dugbe orthonairovirus [DUGV], and HRTV), whereas other viruses were only weakly inhibited or not inhibited at all (such as OROV and Puumala orthohantavirus [PUUV]). IFITM3 has previously been described to inhibit multiple bunyaviruses (36, 52). Exogenous EHD4 also potently inhibited multiple bunyaviruses (including SBV, Batama orthobunyavirus [BMAV], and PUUV), while some viruses were unaffected. Whether this inhibition is physiologically relevant is questionable, as EHD4 is usually expressed at low levels and is only weakly stimulated by IFNs (53, 54). Exogenous HK2 and RHOB caused substantial inhibition in some settings and potently inhibited BUNV and SBV, respectively. However, HK2 and RHOB typically caused only modest inhibition of our panel of viruses.

In contrast to the other ISGs, human and macaque ISG20 potently inhibited the majority of our panel of bunyaviruses (Fig. 3E), in some cases reducing the virus yield >1,000-fold (PUUV and KRIV). Intriguingly, while most bunyaviruses were susceptible to ISG20, Anopheles A orthobunyavirus (ANAV), Capim orthobunyavirus (CAPV), and the three tested phleboviruses (Phenuiviridae family) were largely resistant. ISG20 is an important antiviral exonuclease (37) that has maintained robust interferon stimulation for hundreds of millions of years (54) and has possibly played a key antiviral role throughout this time, with antiviral activity against multiple viruses being documented (37–41). Hitherto, ISG20 has not been ascribed antibunyaviral activity, and the observation that the potent antiviral activity is evaded by multiple viruses suggests that ISG20 directly inhibits specific bunyaviruses (as opposed to inducing global cellular effects). We therefore focused our attention on this ISG.

### Exonuclease activity underpins ISG20-mediated antibunyaviral activity.

**FIG 4:**
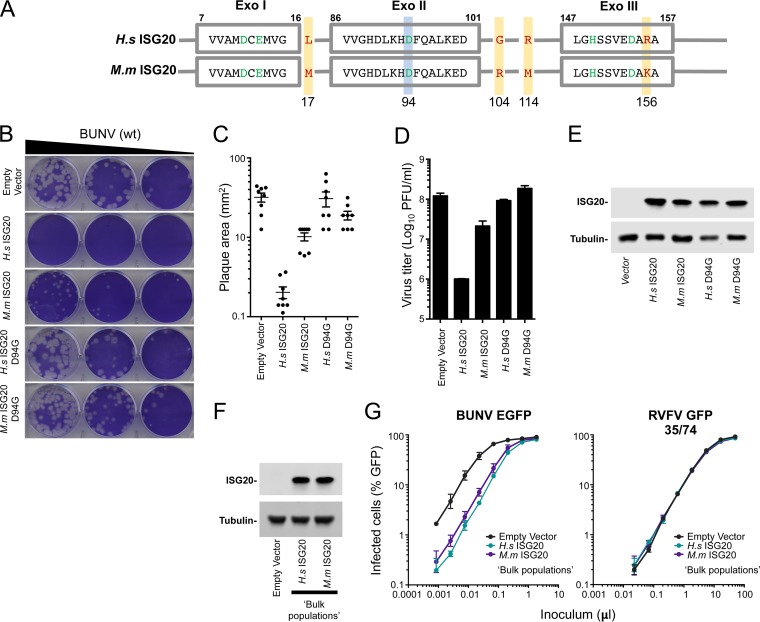
A single point mutation in the active site (ISG20^D94G^) abolishes the antibunyaviral activity of ISG20. (A) Schematic representation of the three exonuclease active motifs (Exo I, II, and III) of ISG20 (shown in gray boxes). The conserved amino acid residues (from the DEDDh family) are highlighted in green. The Asp94 residue (shown in green) of wild-type ISG20 was replaced with Gly to generate the catalytically inactive mutant ISG20^D94G^. The four amino acid differences between the human (H.s) and macaque (M.m) ISG20 proteins are highlighted with yellow boxes and red letters. (B) Plaque phenotypes of wt BUNV on Vero-EMPTY and Vero-ISG20/ISG20^D94G^ cells. (C and D) Quantification of plaque areas (C) and virus titers (D) of BUNV on Vero-EMPTY and Vero-ISG20/ISG20^D94G^ cells. (E) Western blot showing ISG20/ISG20^D94G^ protein expression. Cell lysates were probed with an anti-ISG20 polyclonal antibody. Tubulin served as a sample loading control. (F) Same as panel E, but using bulk populations of Vero cells expressing human and macaque ISG20. (G) Titration curves for the bulk populations from panel F infected with BUNV-EGFP (46) or single-cycle RVFV (57). Means and standard deviations (SD) for triplicate titrations are shown.

Multiple viruses have previously been shown to be sensitive to ISG20, with the majority of inhibition being dependent on ISG20's 3′-to-5′ exonuclease activity (37–41). A single substitution (D94G), part of the DEDDh motif in ISG20's active site (55), can abrogate exonuclease activity and ablate the inhibition of a number of viruses (37–41, 56). To investigate whether exonuclease activity is essential for anti-BUNV activity, we generated cell lines that expressed catalytically inactive forms of human and macaque ISG20 (D94G mutants). While the expression of human and macaque orthologs both reduced the infectious yield of BUNV (by ∼100-fold and ∼10-fold, respectively) and substantially reduced the area of individual BUNV plaques, the catalytically defective mutants had no detectable antiviral activity even though they were expressed at similar levels (Fig. 4B to E).

Our screen of a panel of bunyaviruses indicated that human ISG20 might generally possess more potent antibunyaviral activity than that of the macaque ortholog (Fig. 3 and 4A to E), even though the sequences are 98% identical (at the amino acid level), differing at just 4 amino acid positions (Fig. 4A). Intriguingly, macaque ISG20 also appeared to be more active against CAPV (Fig. 3C), suggesting that ISG20 orthologs might possess divergent antibunyaviral specificities. As Western blot analysis revealed greater expression levels of ISG20 in our human clone (Fig. 3A and 4E), we examined “bulk populations” of Vero cells transduced in parallel with human and macaque ISG20 and found that these bulk populations appeared to express equivalent levels of the ISG20 proteins (Fig. 4F). Importantly, in this context, human and macaque ISG20 inhibited BUNV to similar degrees (Fig. 4G). Crucially, the highly specific antibunyaviral activity of ISG20 was also evident in these bulk populations, as parallel infections with single-cycle RVFV (strain 35/74) (57) were completely unaffected by ISG20 (Fig. 4G). We therefore speculate that any observed species-specific variation in ISG20 activity (Fig. 3C) is perhaps due to different ISG20 expression levels and clonal variation. However, we cannot exclude the possibility that primate ISG20 orthologs may possess divergent antibunyaviral specificities.

### Gene expression from all BUNV genome segments is inhibited by ISG20.

**FIG 5:**
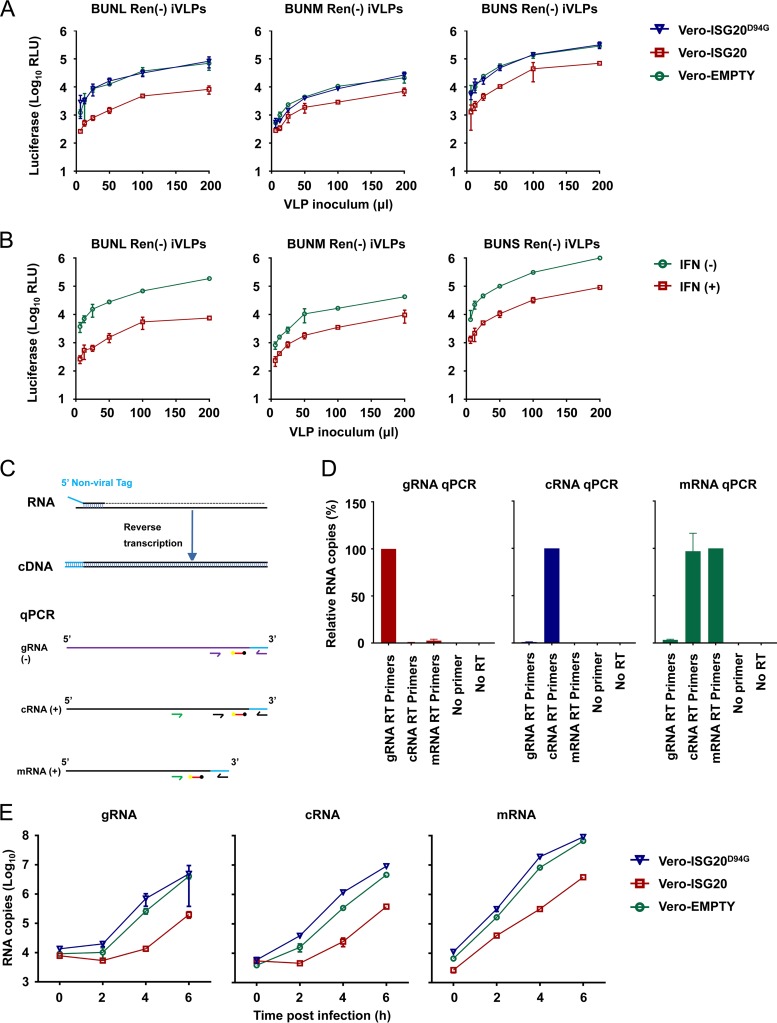
Gene expression from all BUNV genome segments is inhibited by ISG20. (A) Serially diluted single-segment VLPs were used to infect Vero-EMPTY, Vero-ISG20, and Vero-ISG20^D94G^ cells. At 24 hpi, cells were lysed for testing of Renilla luciferase activity (mean ± SD; *n* = 3), and luciferase activity at the indicated VLP inoculum (in microliters) is shown. (B) Antiviral effects of interferon on single-segment VLPs. Unmodified naive Vero cells were treated or mock treated with 1,000 U/ml universal interferon for 24 h. The cells were subsequently infected with 200 μl of serially diluted VLPs. After 24 h, cells were lysed for testing of Renilla luciferase activity (mean ± SD; *n* = 3). (C) RT-qPCR strategies for detection of BUNV S-segment RNAs. Strand-specific tagged reverse transcription primers were used to generate cDNAs for S-segment genomic RNA (gRNA), antigenomic RNA (cRNA), and mRNA, which were subsequently analyzed by qPCR. The mRNA qPCR primers amplify all positive-sense viral RNAs. Using cRNA-specific qPCR primers, mRNA was quantified by subtracting the cRNA abundance from the abundance of all positive-sense viral RNAs. Arrows show qPCR primers, red bars show the TaqMan probe sequence, yellow balls show the 6-carboxyfluorescein (FAM) fluorescent dye, and black balls show Black Hole Quencher 1. (D) Validation of primer specificity. gRNA, cRNA, and mRNA (*in vitro*-transcribed standards) (5 × 10^6^ copies) were reverse transcribed using gRNA, cRNA, and mRNA RT primers, respectively. cDNA was then quantified using strand-specific qPCR primers. (E) Vero-EMPTY or Vero-ISG20/ISG20^D94G^ cells were infected with wt BUNV at an MOI of 10. At 0, 2, 4, and 6 hpi, cells were lysed with TRIzol and total cellular RNA was extracted for RT-qPCR analysis of S-segment RNAs (data are means ± SD; *n* = 3).

BUNV possesses a tripartite (L, M, and S segments), single-stranded, negative-sense RNA genome. To investigate whether ISG20 targets a specific genome segment or all three segments, we assessed the inhibitory effect of ISG20 on virus-like particle (VLP)-based reporter gene expression. In this assay, infectious VLPs (iVLPs) were utilized that contained a single BUNV minigenome derived from just one of the three segments. The iVLPs were assembled in BSRT7/5 cells by supplying all the essential viral proteins (L protein, Gn and Gc viral glycoproteins, and N protein) in *trans* together with a single minigenome transcription vector [either pT7riboBUNLRen(−), pT7riboBUNMRen(−), or pT7riboBUNSRen(−)] representing each genome segment. The minigenomes contained the full-length 5′ and 3′ untranslated regions (UTRs) of BUNV, with the Renilla luciferase gene inserted in place of the viral open reading frame (ORF) in each segment. The iVLPs were used to infect Vero cells expressing either wt ISG20 or ISG20^D94G^. Interestingly, all three minigenome segments were targeted, and luciferase expression from the L, M, and S segments was substantially reduced by ISG20 (Fig. 5A). This ∼5- to 10-fold reduction was entirely dependent on functional exonuclease activity, as the D94G catalytic mutant caused no inhibition of reporter gene expression (Fig. 5A).

We next considered which RNA species might be targeted by ISG20, as multiple RNA forms derived from each genome segment are required for the bunyaviral life cycle. Thus, viral genomic RNA (gRNA), antigenomic RNA (cRNA), and mRNA species could all conceivably be targets of ISG20. We were not aware of existing bunyaviral strand-specific quantitative PCR (qPCR) assays, so based on published norovirus and influenza A virus strategies (58, 59), we developed a tagged TaqMan quantitative reverse transcription-PCR (qRT-PCR) assay that efficiently detects the desired S-segment RNA species but poorly amplifies undesired/nonspecific targets. The exception was mRNA, whose entire sequence is also present in the cRNA species, except for the “cap-snatched,” host cell-derived 5′ end (Fig. 5C). In this instance, we estimated the mRNA copy number by subtracting the cRNA copy number from the value obtained from the mRNA assay (which detects all positive-sense viral RNAs). Validation of the assay specificity is shown in Fig. 5D. Since a single cycle of BUNV infection in Vero cells is approximately 6 h (46), we examined the S segment at 0, 2, 4, and 6 h postinfection. Using this approach, all three S-segment RNAs appeared to be diminished substantially (∼10-fold) by ISG20 following infection of Vero cells (Fig. 5E). However, we were unable to discern whether this reduction was due to the S-segment RNAs being degraded by ISG20 or was an indirect consequence of ISG20 inhibiting some other facet(s) of the BUNV life cycle.

### Mapping the viral determinants of ISG20 antiviral activity.

**FIG 6:**
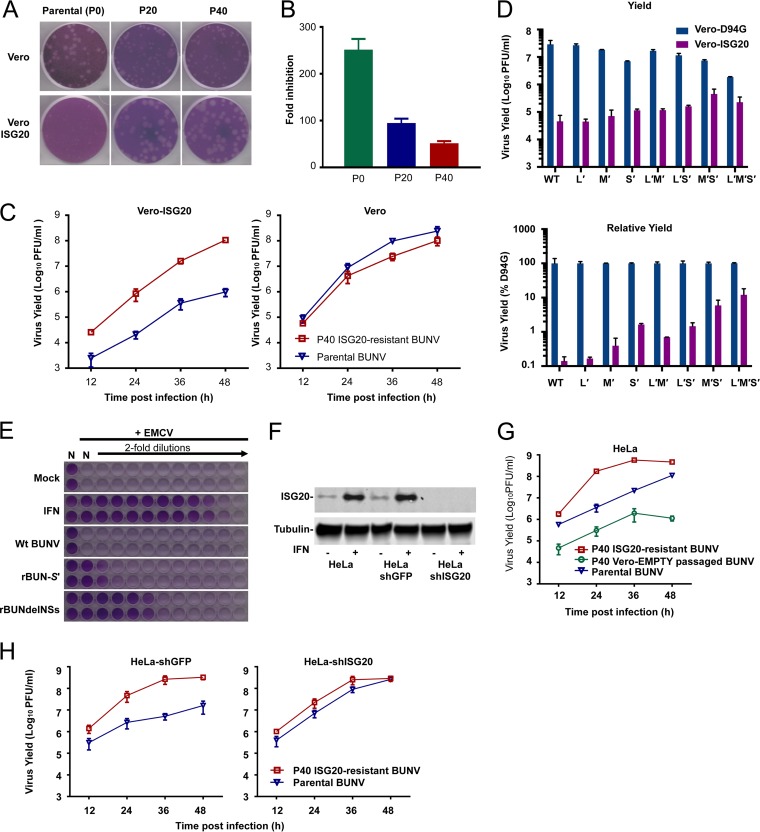
Generation of ISG20-resistant BUNV and demonstration that endogenous ISG20 possesses antibunyaviral activity. (A and B) Plaque phenotypes (on Vero and Vero-ISG20 cells) (A) and fold inhibition (B) of the parental virus and the P20 and P40 ISG20-resistant viruses by ISG20 (fold reductions in PFU per milliliter for comparisons of naive and Vero-ISG20 cells). (C) Comparison of growth curves for the parental virus and the P40 virus on Vero-ISG20 and naive Vero cells. Cells were infected at an MOI of 0.01 (data are means ± SD; *n* = 3). (D) Infectious yields (36 h after infection at an MOI of 0.01) of wild-type (wt) BUNV and reassortant mutants (mutant segments are denoted by “′”) from Vero-ISG20 or Vero-D94G cells (data are means and ranges; *n* = 2). (E) Biological IFN assays to assess the ability of supernatants from HeLa cells infected with the indicated viruses to inhibit the replication of EMCV in A549-Npro cells. (F) Western blot analysis of basal and inducible expression in naive HeLa cells, HeLa-shGFP cells, and HeLa-shISG20 cells. Basal expression or universal interferon (500 U/ml)-induced ISG20 expression was stably detected in naive HeLa cells and HeLa-shGFP cells, whereas both basal and inducible ISG20 expression was undetectable in HeLa-shISG20 cells. (G) Growth curves for parental BUNV, P40 Vero-EMPTY-passaged BUNV, and P40 ISG20-resistant BUNV on HeLa cells (MOI = 0.01) (data are means ± SD; *n* = 3). (H) Growth curves for parental BUNV and P40 ISG20-resistant BUNV on HeLa-shGFP (control) and HeLa-shISG20 cells.

*In vitro* evolution/serial passaging has often been employed to study the antiviral mechanisms of many antiviral factors and frequently reveals the sensitivity determinants that represent a molecular interface between host and pathogen (60–64). To investigate whether BUNV can access sequence space that confers resistance to ISG20, we passaged BUNV in Vero cells expressing human ISG20 (or modified using empty vector). We periodically monitored the ability of the passaged swarm to plaque/replicate in the presence of ISG20. Following 20 passages, partial ISG20 resistance was observed, and after 40 passages in the presence of ISG20, we were able to isolate a viral swarm that replicated far more efficiently in the presence of ISG20, forming larger plaques and yielding more infectious particles (Fig. 6A to C). This P40 virus replicated substantially faster than the parental virus in the presence of ISG20, yielding ∼100-fold more virus at 48 hpi (Fig. 6C). Crucially, the P40 virus was weakly attenuated on naive Vero cells, suggesting that the ISG20 resistance was not merely due to adaptation to Vero cells during the passage series (Fig. 6C).

To investigate the viral determinants that rendered BUNV resistant to ISG20, we sequenced the L, M, and S segments of the P20 and P40 swarms by using direct sequencing of RT-PCR products. The sequences of the termini were also simultaneously determined using 3′ rapid amplification of cDNA ends (RACE). Sequence analysis revealed mutations in all three segments at P20 and P40, some of which were transient polymorphisms. By P20, apart from a reversion to the GenBank sequence (A3568G; M segment), only a single mutation was selected to near uniformity in the presence of ISG20 (Table 3). This S-segment mutation (A330C) encodes changes in the N (N82T) and NSs proteins (T76P) and is possibly the major determinant of the ISG20 resistance observed at P20. Sequencing of the P40 swarm revealed multiple mutations in all genome segments that had been selected to near uniformity (Table 3). Importantly, none of these mutations were found in the P40 virus passaged on Vero-EMPTY cells (Table 4).

**TABLE 3:** Nucleotide and amino acid changes of BUNV passaged on Vero-ISG20 cells^*a*^

Segment	Position (nt)	Nucleotide mutation			Amino acid mutation	Note
		P0	P20	P40		
L	3,257	G	G	A	Synonymous	
	5,267	A	A	G	I1740M	L protein
	6,538	A	A/G^*b*^	G	E2163A	L protein
M	518	T	T/A^*b*^	T	Synonymous	
	1,371	G	G/A^*b*^	G	V439I	NSm
	3,568	A	G^*c*^	G^*c*^	N1171S	Gc protein
	4,388	A	A/G^*b*^	G	None	cRNA 3′ UTR
S	112	C	C/T^*b*^	T	Synonymous	N protein
					S3L	NSs protein
	330	A	C	C	N82T	N protein
					T76P	NSs protein

a The following differences were observed in the P0 virus relative to the sequence in GenBank: for the L segment (accession number NC_001925.1), T5501C (silent) mutation and 6,809-nucleotide C insertion in the 5′ UTR; for the M segment (accession number NC_001926.1), G415A (R120K), A417G (K121E), G1423A (R456K), A1941G (S629G), C2638T (P861L), G2829A (E925K), T3359C (silent), G3568A (S1171N), A4008G (R1381G), and G4317A (A1421T); and for the S segment (accession number NC_001927.1), not available.

b Polymorphism.

c Reversion to the GenBank sequence.

**TABLE 4:** Nucleotide and amino acid changes of BUNV passaged on Vero-EMPTY cells^*a*^

Segment	Position (nt)	Nucleotide mutation			Amino acid mutation	Note
		P0	P20	P40		
L	3,299	A	A	T	Synonymous	
	3,622	A	A	A/G^*b*^	E1191G	L protein
M	518	T	T/A^*b*^	T	Synonymous	
	1,371	G	G/A^*b*^	G	V439I	NSm
	2,500	C	T	T	P815L	Gc protein
	4,240	T	T/A^*b*^	A	I1395N	Gc protein
S	84	T	T	C	None	3′ UTR
	118	T	T	C	Synonymous	N protein
					L5P	NSs protein
	165	A	G	G	K27R	N protein
					S21G	NSs protein

a The following differences were observed in the P0 virus relative to the sequence in GenBank: for the L segment (accession number NC_001925.1), T5501C (silent) mutation and 6,809-nucleotide C insertion in the 5′ UTR; for the M segment (accession number NC_001926.1), G415A (R120K), A417G (K121E), G1423A (R456K), A1941G (S629G), C2638T (P861L), G2829A (E925K), T3359C (silent), G3568A (S1171N), A4008G (R1381G), and G4317A (A1421T); and for the S segment (accession number NC_001927.1), not available.

b Polymorphism.

To assess the significance of these mutations, we generated reassortant viruses that contained parental and P40 mutant genome segments [the “′” symbol denotes the mutant segment(s) used] by using reverse genetics. Importantly, a virus rescued with all three P40 genome segments (designated rBUN-L′M′S′) recapitulated the phenotype of the P40 swarm and replicated more efficiently than the wt virus in the presence of ISG20 (Fig. 6D). In accordance with the observation that ISG20 inhibits gene expression from all three genome segments (Fig. 5A), the addition of a single P40 genome segment was not sufficient to confer equivalent ISG20 resistance to the L′M′S′ virus (Fig. 6D). Moreover, the contribution of each individual mutant segment to ISG20 resistance appeared to be unequal, with S′ and M′ conferring more substantial resistance than that seen with L′. Thus, ISG20 resistance appears to be multipartite, and mutations are required on more than one genome segment for the P40 virus to resist ISG20. Because the mutant S′ segment encoded two substitutions in NSs (S3L and T76P), we wondered whether the mutant NSs protein still functioned as an interferon antagonist. Interestingly, the mutant NSs protein appeared to have reduced activity as an IFN antagonist in an encephalomyocarditis virus (EMCV) inhibition assay (Fig. 6E).

### Endogenous ISG20 has anti-BUNV activity.

A recurrent obstacle in understanding how the IFN-induced “antiviral state” inhibits a virus is that viruses are often simultaneously inhibited by multiple IFN-induced defenses. In this scenario, loss-of-function experiments can be challenging, as viral replication might not be rescued substantially by removing a single ISG (because multiple inhibitory effectors might still be fully active). Thus, we searched for cell lines that expressed readily detectable ISG20 without the need for prior stimulation. HeLa cells are known to express ISG20 in the absence of IFN treatment (37, 38) (Fig. 6F). Accordingly, the ISG20-resistant swarm grew far more efficiently than the parental virus in unmodified HeLa cells, with the P40 virus yielding ∼100-fold more virus at 24 h postinfection (Fig. 6G). However, the control P40 virus (Vero-EMPTY) did not exhibit any growth advantage on unmodified HeLa cells (Fig. 6G, green line). Crucially, this difference between the P40 ISG20-resistant virus and the parental virus was largely due to endogenous ISG20, as short hairpin RNA (shRNA)-mediated knockdown of ISG20 increased the replication of the parental virus so that it replicated with kinetics similar to those of the P40 virus (Fig. 6H). Thus, even low levels of endogenous ISG20 can cause substantial inhibition of BUNV, suggesting that IFN-induced upregulation of ISG20 (Fig. 6F) likely represents a formidable block to the replication of BUNV and other sensitive bunyaviruses.

## DISCUSSION

Interferons rapidly engender an “antiviral state” in host cells through the induction and expression of hundreds of ISGs, many of which have antiviral functions. It is well documented that interferons can suppress the replication of divergent bunyaviruses both *in vitro* and *in vivo* (9–15). In this study, we screened 488 ISGs and identified multiple ISGs that inhibited the prototypical bunyavirus (BUNV). We revealed that ISG20 exhibits potent and highly specific antibunyaviral activity. ISG20 is robustly upregulated in response to interferons, and the ISG20 promoter contains ISRE and GAS elements that respond to type I, type II, and type III interferons (38, 65). Multiple studies have highlighted the importance of ISG20 as an antiviral effector that targets multiple viruses (37–41), and several bunyaviruses can be added to the growing list of ISG20 targets. Thus, ISG20-mediated inhibition may constrain bunyaviral replication as part of an interferon response, and this targeted activity of ISG20 may limit the pathogenesis of specific bunyaviruses.

We previously screened the ability of over 350 human ISGs to inhibit BUNV in Huh7 cells and did not identify any candidates that inhibited BUNV >2-fold (42). Nonetheless, the most inhibitory ISGs (IFITM3 and IRF1) were reidentified in this study. Crucially, ISG20 has been reported to lack specific antiviral activity in Huh7 cells (41), potentially explaining why ISG20 was not ascribed antibunyaviral activity in previous screens. Moreover, the unanticipated single round of BUNV-EGFP infection in MT4 cells likely increased the fidelity of the primary screen, facilitating the identification of multiple candidate effectors in this study.

A few ISGs previously reported to inhibit BUNV (PKR, IFI44, and Viperin) (32) were not reidentified in our primary screen. Notably, we were unable to achieve efficient transduction with PKR-encoding SCRPSY vectors (43), possibly explaining the lack of antiviral activity in this primary screen. Akin to ISG20 in Huh7 cells, multiple ISGs possess cell-type-dependent activity. Accordingly, parallel screens with different cell lines can identify different antiviral candidates (43). Thus, the anti-BUNV activity of IFI44 and Viperin may be context dependent or might simply block the virus life cycle after efficient GFP expression is achieved (further work would be necessary to explore either possibility).

The antiviral activity of ISG20 appeared to rely on its 3′-to-5′ exoribonuclease function, as a single amino acid substitution within the active site (D94G) abrogated antiviral activity. However, it is possible that disrupting this coordinating residue grossly alters the structure of ISG20, placing a slight caveat on this interpretation. In contrast to RNase L, an interferon-stimulated RNase that indiscriminately cleaves single-stranded RNA following UU and UA motifs, ISG20 is able to discriminate between self RNAs (rRNAs [38, 39] and β-actin mRNA [40]) and nonself (viral) RNAs. Similarly, we observed that Vero cells expressing exogenous ISG20 appeared to be healthy (38) and that plasmid-based expression was equally efficient in Vero-ISG20 and control cell lines (our unpublished observations). Nonetheless, it seems likely that host RNAs do not completely escape ISG20 because, in accordance with previous observations (56), Vero cells modified to express ISG20 proliferated more slowly than unmodified cells (unpublished observations). Importantly, the fact that all the phleboviruses we tested (RVFV strains MP12 and 35/74, SFTSV, and, to a lesser extent, HRTV) and two orthobunyaviruses (CAPV and, to a lesser extent, ANAV) largely escaped ISG20-mediated inhibition underscores the high level of target specificity exhibited by ISG20. Whether these resistant viruses actively antagonize ISG20 function (by deploying viral countermeasures) or evade inhibition through minimizing sequence/structural motifs targeted by ISG20 is worthy of future investigation. We currently have no information on whether bunyaviral protein or RNA is predominantly involved, and the precise viral sensitivity/resistance determinants that underlie ISG20's selective antibunyaviral activity are completely unknown (and may influence the pathogenicity of bunyaviruses).

Interestingly, although ISG20 is highly specific, all three luciferase-encoding minigenomes (reminiscent of each BUNV genome segment) were targeted by ISG20. The VLP system we used can complete only a single round of infection yet was potently inhibited by ISG20. Thus, ISG20 may represent a rapid and effective block to the bunyavirus life cycle. Crucially, viral replication and transcription in the VLP-infected cells were carried out by ribonucleoproteins (RNPs) packaged into the VLPs, in which only one minigenome species (representing a specific genome segment) was present. Therefore, efficient reporter gene expression was not dependent upon gene expression from other genome segments, and under these conditions, ISG20 could not mediate indirect inhibition by targeting another genome segment. Thus, it is likely that following VLP infection, ISG20 directly targeted the L, M, and S minigenome segments. One possible caveat is that the minigenome segments are considerably shorter than the wt segments and therefore might not fully recapitulate the ISG20 sensitivity/resistance of the complete segments. Moreover, internal RNA structures can govern sensitivity/resistance to ISG20 inhibition (38), and we cannot rule out the possibility that the luciferase ORF constitutes a particularly attractive target for ISG20.

Because BUNV genome replication and gene expression are tightly coupled, it is unclear whether minigenome gRNA, cRNA, mRNA, or a combination of these species is targeted by ISG20. Using qRT-PCR, we further examined the S-segment RNA in the context of replication-competent unmodified BUNV. The quantification of three S-segment RNAs (gRNA, cRNA, and mRNA) indicated that the abundances of all three were decreased by ISG20. Considering the VLP data and these qPCR data together, it seems likely that S-segment RNAs are directly targeted by ISG20. However, we cannot exclude the possibility that the observed reduction in S-segment RNAs was an indirect consequence of reduced viral polymerase activity caused by ISG20 inhibiting another aspect of the BUNV life cycle.

Development of an ISG20-resistant BUNV through *in vitro* evolution revealed multiple viral determinants of ISG20 antiviral sensitivity/resistance. The mutations required for ISG20 resistance did not map to a single genome segment, and instead, changes were observed in all three segments. Although individual mutant segments could increase virus growth in the face of ISG20, no single mutant segment could recapitulate the considerable resistance observed using all three mutant segments. This polygenic resistance profile considered with the sensitivity of all three minigenomes to ISG20-mediated inhibition suggests that multiple BUNV RNA segments are likely targets of ISG20. We speculate that if all genome segments are targeted by ISG20, then mutations of each segment may plausibly make these viral RNAs less attractive substrates for ISG20. Notably, the only M-segment mutation selected in the presence of ISG20 was in a noncoding region (A4388G), excluding reversion to the GenBank sequence. This observation suggests that BUNV RNA sequence or structural elements might define sensitivity/resistance to ISG20. However, we cannot exclude a role for viral proteins in conferring resistance to ISG20. Notably, the S′ segment conferred the most substantial resistance to ISG20, and by P20, a substitution in the N protein (N82T) had been selected to near uniformity. Because the N protein forms a complex with RNAs from all segments, it is possible that the N protein influences ISG20 sensitivity. However, in the only instance where we assessed protein function (derived from the ISG20-resistant virus), the mutant NSs protein appeared to be less potent than the parental protein as an interferon antagonist. Although this is not evidence for RNA-based ISG20 resistance, it is concordant with this possibility. Put simply, the mechanistic basis for how the P40 virus (or any bunyavirus) resists ISG20 is unknown. Moreover, while progress has been made (38, 56), the molecular features that define RNAs as sensitive or resistant to ISG20-mediated inhibition (underlying self-nonself discrimination by ISG20) have yet to be elucidated fully.

In summary, we have revealed that ISG20 inhibits specific bunyaviruses and have demonstrated that endogenous ISG20 can potently inhibit BUNV replication. Our study reinforces the notion that ISG screening is a powerful method for identifying antiviral effectors. Moreover, the molecular basis of how interferons constrain the replication of an important group of viral pathogens and how some viruses escape this inhibition may have impacts on the pathogenesis of bunyaviruses of clinical significance, many of which cause life-threatening acute febrile illnesses.

## MATERIALS AND METHODS

### Cells and viruses.

MT4 cells were maintained in RPMI 1640 medium (Gibco) supplemented with 9% fetal bovine serum (FBS) and 100 μg/ml gentamicin. BHK-21 cells and BSRT7/5 cells (modified to stably express T7 RNA polymerase) (66) were grown in Glasgow minimum essential medium (GMEM; Thermo Fisher Scientific) supplemented with 9% newborn bovine serum (NBS) and FBS, respectively, in addition to 10% tryptose phosphate broth. BSR-T7/5 cell medium was additionally supplemented with 1 mg/ml of Geneticin (G418) sulfate (Calbiochem). Vero-E6 (referred to as Vero cells throughout), HeLa, HEK 293T, and A549-Npro cells (expressing the bovine viral diarrhea virus NPro protein) (67) were maintained in Dulbecco's modified Eagle medium (DMEM; Thermo Fisher Scientific) supplemented with 9% FBS. HEK 293T cell medium was also supplemented with 100 μg/ml gentamicin.

Fifteen bunyaviruses were used in the study, including BUNV, Cache Valley virus (CVV), Kairi virus (KRIV), Anopheles A virus (ANAV), Tacaiuma virus (TCMV), Boraceia virus (BORV), Capim virus (CAPV), Oropouche virus (OROV), Schmallenberg virus (SBV), Batama virus (BMAV), Puumala virus (PUUV), RVFV (MP12), SFTSV, and heartland virus (HRTV). rBUN-Gc-EGFP (called BUNV-EGFP here) is a recombinant BUNV in which truncated Gc is tagged with EGFP (46). The single-cycle RVFV system was described previously (57).

### Plasmids.

The SCRPSY lentiviral vector (GenBank accession number KT368137) and the human and macaque ISG libraries have been described previously (43–45). An shRNA-encoding lentiviral vector targeting ISG20 was generated by PCR, using the human U6 promoter as a template and the following oligonucleotides: 5′-CTCTCTGAATTCCTGCAGAAGGTCGGGCAGGAAGAGGGCCTA-3′ and 5′-TCTCTCGAATTCAAAAAGGTGCTGTGCTGTACGACAAGTTCGAAAACTTATCGTACAGCACAGCACCGGTGTTTCGTCCTTTCCACAAGATATATAAAG-3′. The PCR product was inserted into CSPW by use of EcoRI (68). The GFP shRNA has been described previously (68). Plasmids pTM1BUNL, pTM1BUNM, and pTM1BUNN, encoding BUNV proteins L, M (polyprotein precursor), and N, respectively, and pT7riboBUNLRen(−), pT7riboBUNMRen(−), and pT7riboBUNSRen(−), used to generate viral genomic-sense minigenomes, have been described previously (69). Full-length cDNA sequences of the L, M, and S segments were subcloned into plasmid pTVT7R (0,0) (70) to generate three rescue plasmids: pTVT7 BUNL(+), pTVT7 BUNM(+), and pTVT7 BUNS(+).

### FACS-based screening.

The fluorescence-activated cell sorter (FACS)-based screening assay was performed in a fashion similar to that for previously described screens (43–45). Briefly, two lentiviral vector-encoded ISG libraries, consisting of 488 unique ISGs, including 394 human ISGs and 344 macaque ISGs (43), were used to transduce MT4 cells. Each ISG was expressed from the completely spliced “early” HIV-1 mRNA, while TagRFP (a surrogate marker for transduction) was expressed from an unspliced late HIV-1 mRNA (43). This vector format was previously validated, ensuring that RFP-expressing cells were also ISG positive (using EGFP inserted in place of the ISG) (unpublished observations).

Target MT4 cells were transduced with lentiviral vectors encoding the human and macaque ISGs and incubated for 48 h (to allow ISG expression) prior to challenge with BUNV-EGFP at an input equivalent to a multiplicity of infection (MOI) of 5 (Fig. 1C), determined using Vero cells. This dose resulted in an average of ∼35% infection. At 48 hpi, cells were collected and fixed with 4% formaldehyde. The percentage of transduced cells that were infected with BUNV-EGFP was determined by flow cytometry using a Guava EasyCyte flow cytometer (Millipore).

### Lentiviral vector production and transduction assays.

Lentiviral stocks were generated in 293T cells by cotransfection of the SCRPSY lentiviral plasmid, a plasmid expressing HIV-1 NL4-3 *gag-pol* (pNLGP), and a plasmid expressing the vesicular stomatitis virus glycoprotein (pVSV-G) at a ratio of 250 ng:25 ng:5 ng in a 96-well plate (for ISG library production) or 5 μg:5 μg:1 μg in a 10-cm dish (for confirmatory assays). The SCRPSY lentiviral plasmid carries a puromycin resistance gene in a single ORF together with TagRFP, separated by a foot-and-mouth disease virus (FMDV) 2A “ribosomal skipping” peptide (PAC2ATagRFP; GenBank accession number ANN89731.1) that facilitated puromycin selection of transduced cells. Supernatants from 10-cm dishes were collected at 48, 72, and 96 h posttransfection, clarified by filtration through a 0.22-μm filter, and stored at −80°C. For transduction assays, MT4 or Vero cells were seeded in either 96-well (40,000 cells/well) or 24-well (120,000 cells/well) plates. Vero cells were transduced with lentiviral vectors by spinoculation at 1,600 rpm for 1 h. For confirmatory experiments, new lentiviral stocks were generated for selected ISGs, and the inhibitory effect was measured through triplicate titrated challenges fixed at 48 hpi (BUNV-EGFP) or 24 hpi (RVFV-EGFP) (57). The ISG-expressing Vero cells were selected and maintained in medium containing 10 μg/ml puromycin.

### Virus infection and titration.

For assays of infectious yields, cells were seeded in either 12-well or 6-well plates and infected using MOIs of 0.1 to 0.001 (see the figure legends for specific values). The infected cell culture supernatants were harvested after incubation for 48 or 72 h. Virus titers were determined by plaque assay. Briefly, cell monolayers were infected with serially diluted virus, incubated for 1 h at 37°C, and then covered with a 0.6% Avicel (FMC Biopolymer)-minimum essential medium (MEM) overlay medium supplemented with 2% NBS or FBS. After incubation for 3 to 11 days, depending on the respective virus, cells were fixed with 4% formaldehyde–phosphate-buffered saline (PBS) and stained with 0.5% (wt/vol) methyl violet to visualize the plaques. Virus titers were calculated and presented as numbers of PFU per milliliter. For PUUV only, the foci were visualized using an immunofocus assay as described previously (4).

### Infectious VLP assays.

VLP assays were performed as described previously (71), with minor modifications. In brief, BSRT7/5 cells grown in 6-well plates were transfected with plasmids pTM1-BUNVN (0.2 μg), pTM1-BUNL (0.3 μg), and pTM1-BUNM (0.2 μg) and a BUNV-derived minigenome plasmid (0.4 μg) [pT7riboBUNLRen(−), pT7riboBUNMRen(−), or pT7riboBUNSRen(−)] by use of TransIT-LT1 transfection reagent (Mirus). At 24 h posttransfection, supernatants were harvested and clarified at 4,000 rpm for 3 min. Each VLP-containing supernatant was serially diluted and used to infect Vero-ISG cells seeded in 96-well plates. Renilla luciferase activity was measured after a further 24 h of incubation by using a dual-luciferase assay kit (Promega) according to the manufacturer's instructions.

### Western blotting.

Cell lysates were prepared by the addition of SDS sample buffer (20% glycerol, 100 mM Tris-HCl [pH 6.8], 4% SDS, 200 mM dithiothreitol [DTT], 0.2% bromophenol blue). Proteins were then separated in NuPage 4 to 12% Bis-Tris gradient polyacrylamide gels (Nu-PAGE; Life Technologies) and wet transferred to a nitrocellulose blotting membrane (GE Healthcare). The membrane was blocked in Sea Block blocking buffer (Thermo Scientific) for 2 h at room temperature and then probed with a rabbit anti-human ISG20 polyclonal antibody (PA5-30073; Thermo Fisher Scientific) and a mouse anti-tubulin monoclonal antibody (T5168; Sigma) at room temperature for 2 h. After washing with PBS-Tween 20, the membrane was subsequently probed with fluorescent secondary antibodies, i.e., IRDye 680RD-labeled goat anti-rabbit antibody (Thermo Scientific) and IRDye 800CW-labeled goat anti-mouse antibody (Li-Cor Biosciences). The blots were scanned using a quantitative Li-Cor Odyssey scanner. The blot shown in Fig. 3A was created as described previously (4), using antibodies raised against EHD4 (ab153892), SCO2 (ab169042), and LGALS9 (ab153673) from Abcam in addition to antibodies against HK II (sc-130358), IFTM3 (sc-100768), IRF1 (Sc-135952), NOS2A (Hs sc-7271), and RHOB (sc-8048) from Santa Cruz. We were unable to establish a Western blot assay for NOS2A or RHOB.

### Virus *in vitro* evolution.

Vero-ISG20 cells in T25 flasks were initially infected with the parental BUNV strain at an MOI of 0.01. After 48 h, the supernatant (passage 1) was used to infect a fresh batch of Vero-ISG20 cells. Subsequent passages were performed up to passage 40, using an MOI of ∼0.01. A parallel passage series was completed using Vero-EMPTY cells (transduced with an “empty” SCRPSY vector) as the substrate, also for 40 passages to monitor adaptation to transduced Vero cells. The supernatants were collected and stored at −80°C, and virus titers were determined by plaque assay on Vero cells.

### Virus genome sequencing and 3′-RACE.

To determine the genomic sequences of the parental BUNV and ISG20-resistant BUNV strains, Vero and Vero-ISG20 cells were infected with parental BUNV and ISG20-resistant BUNV, respectively, at an MOI of 0.1. Cells were lysed in TRIzol (Invitrogen) at 24 hpi, and total cellular RNA was extracted in accordance with the manufacturer's instructions. The viral RNA was subsequently amplified by RT-PCR, using GoScript reverse transcriptase (Promega) and Q5 high-fidelity DNA polymerase (New England BioLabs), and the sequence was determined by direct sequencing of the PCR products (Eurofins Genomics).

The sequences of the termini of the viral segments were determined by 3′-RACE. Extracted RNA was polyadenylated using a poly(A) tailing kit (Ambion) to generate 3′-poly(A)-tailed genomic and antigenomic RNAs. cDNA was prepared using oligo(dT). The 3′ termini were then PCR amplified using oligo(dT) (Ambion) and a sequence-specific internal primer. The sequence of the 5′ UTR of the genomic RNA was determined by sequencing the 3′ UTR of the antigenomic RNA (as described above). The amplified fragments were gel purified, and the sequence was determined by Eurofins Genomics.

### BUNV S-segment qRT-PCR.

Vero-EMPTY, Vero-ISG20, and Vero-ISG20^D94G^ cells were infected with BUNV at an MOI of 10. At 0, 2, 4, and 6 hpi, cells were lysed in TRIzol and total cellular RNA was extracted as described above. Following quantification using a NanoDrop 2000 spectrophotometer (Thermo Scientific), RNAs were reverse transcribed into cDNAs by use of strand-specific tagged RT primers and a SuperScript III first-strand synthesis system. RNA standards generated from *in vitro*-transcribed viral genomic RNA (gRNA), antigenomic RNA (cRNA), and mRNA were used as quantification standards. Quantitative PCR was performed using Brilliant III Ultra-Fast QPCR master mix from Agilent Technologies and a Stratagene Mx3005P platform from Agilent Technologies. Each qPCR was performed with a 20-μl reaction volume (6.2 μl distilled water [dH_2_O], 10 μl 2× master mix, 0.5 μl forward oligonucleotide [10 μM], 0.5 μl reverse oligonucleotide [10 μM], 0.5 μl probe [5 μM], 0.3 μl ROX [1:500 dilution], and 2 μl cDNA template). The qPCR cycling parameters were 95°C for 3 min followed by 40 cycles of 95°C for 10 s and 60°C for 22 s. Each reaction was performed in triplicate. Details of the RT-qPCR primers are listed in Table 5.

**TABLE 5:** Primers used for BUNV S-segment qRT-PCRs^*a*^

Reaction	Target and primer or probe	Oligonucleotide sequence (5′ → 3′)	Position on RNA segment
RT	gRNA	*GGCCGTCATGGTGGCGAAT*CTTGCTATTGTTGAAAATCGCTGTGCTA	911–938
	cRNA	*GCTAGCTTCAGCTAGGCATC*CAATATAATGTTGATTTAGCCC	903–924
	mRNA	*CCAGATCGTTCGAGTCGT*CATCCCTGCTTACATGTTGATTC	774–796
qPCR	gRNA forward primer	CGACATCATGAAATTCCAA	849–867
	gRNA probe	FAM-ATCCAACAGAAGGTCATTAAAGGCTC-BHQ1	881–906
	gRNA reverse primer	*GGCCGTCATGGTGGCGAAT*	
	cRNA forward primer	ACAAAATAACAGCTGCTTGG	856–875
	cRNA probe	FAM-TTTAGCCCGCTGTCTTTCTGTCCC-BHQ1	887–910
	cRNA reverse primer	*GCTAGCTTCAGCTAGGCATC*	
	mRNA forward primer	CAGTTGTCTCTAGCTTAGGTTGG	702–724
	mRNA probe	FAM-TCCCTGGCAGCTGCACTAACATT-BHQ1	734–756
	mRNA reverse primer	*CCAGATCGTTCGAGTCGT*	

a Based on the S-segment sequence under GenBank accession number NC_001927.1. Italic sequences show the nonviral tags.

### Virus rescue using reverse genetics.

Virus rescues were performed as described previously (72), with minor modifications. In brief, subconfluent BSRT7/5 cells were transfected with a mixture of plasmids comprising 1.0 μg each of either wild-type or mutant (specified in the relevant figure legends) pTVT7 BUNL(+), pTVT7 BUNM(+) and pTVT7 BUNS(+) and 9 μl of TransIT-LT1 transfection reagent (Mirus Bio LLC) in 0.7 ml of Opti-MEM medium (Life Technologies). After 4 h of transfection, 4 ml of growth medium was added, and incubation was continued for 4 days or until cytopathic effect (CPE) was evident. Supernatant was harvested and stored at −80°C.

### Biological interferon assay.

The biological assay for IFN production was performed as described previously (73). Briefly, “IFN-competent” HeLa cells were infected with different BUNV mutants (MOI = 3) and incubated at 37°C for 24 h. The supernatant was clarified by a brief centrifugation and inactivated by UV irradiation. The UV-inactivated supernatant was 2-fold serially diluted and used to treat “IFN-incompetent” A549-Npro cells (expressing the bovine viral diarrhea virus NPro protein). Cells were infected with encephalomyocarditis virus (EMCV) at an MOI of 0.01, fixed with 8% formaldehyde, and stained with a methyl violet staining solution (5% [wt/vol]).
